# Is Artificial Intelligence the Cost-Saving Lens to Diabetic Retinopathy Screening in Low- and Middle-Income Countries?

**DOI:** 10.7759/cureus.45539

**Published:** 2023-09-19

**Authors:** Anza Rizvi, Fatima Rizvi, Parth Lalakia, Leslie Hyman, Rosemary Frasso, Les Sztandera, Anthony Vipin Das

**Affiliations:** 1 Sidney Kimmel Medical College, Thomas Jefferson University, Philadelphia, USA; 2 College of Population Health, Thomas Jefferson University, Philadelphia, USA; 3 Osteopathic Medicine, Rowan-Virtua School of Osteopathic Medicine, Stratford, USA; 4 Office of Global Affairs, Thomas Jefferson University, Philadelphia, USA; 5 Geriatric Medicine and Palliative Care, Department of Family Medicine, Thomas Jefferson University, Philadelphia, USA; 6 The Vickie and Jack Farber Vision Research Center, Wills Eye Hospital, Philadelphia, USA; 7 Asano-Gonnella Center for Research in Medical Education and Health Care, Thomas Jefferson University, Philadelphia, USA; 8 Kanbar College of Design, Engineering, and Commerce, Thomas Jefferson University, Philadelphia, USA; 9 Ophthalmology, LV Prasad Eye Institute, Hyderabad, IND

**Keywords:** health economic, diabetic retinopathy screening, low- and middle-income countries, ophthalmology, ai & robotics in healthcare

## Abstract

Diabetes is a rapidly growing global health crisis disproportionately affecting low- and middle-income countries (LMICs). The emergence of diabetes as a global pandemic is one of the major challenges to human health, as long-term microvascular complications such as diabetic retinopathy (DR) can lead to irreversible blindness. Leveraging artificial intelligence (AI) technology may improve the diagnostic accuracy, efficiency, and accessibility of DR screenings across LMICs. However, there is a gap between the potential of AI technology and its implementation in clinical practice. The main objective of this systematic review is to summarize the currently available literature on the health economic assessments of AI implementation for DR screening in LMICs. The review was conducted according to the Preferred Reporting Items for Systematic Reviews and Meta-Analysis (PRISMA) guidelines. We conducted an extensive systematic search of PubMed/MEDLINE, Scopus, and the Web of Science on July 15, 2023. Our review included full-text English-language articles from any publication year. The Joanna Briggs Institute's (JBI) critical appraisal checklist for economic evaluations was used to rate the quality and rigor of the selected articles. The initial search generated 1,423 records and was narrowed to five full-text articles through comprehensive inclusion and exclusion criteria. Of the five articles included in our systematic review, two used a cost-effectiveness analysis, two used a cost-utility analysis, and one used both a cost-effectiveness analysis and a cost-utility analysis. Across the five articles, LMICs such as China, Thailand, and Brazil were represented in the economic evaluations and models. Overall, three out of the five articles concluded that AI-based DR screening was more cost-effective in comparison to standard-of-care screening methods. Our systematic review highlights the need for more primary health economic analyses that carefully evaluate the economic implications of adopting AI technology for DR screening in LMICs. We hope this systematic review will offer valuable guidance to healthcare providers, scientists, and legislators to support appropriate decision-making regarding the implementation of AI algorithms for DR screening in healthcare workflows.

## Introduction and background

Diabetes presents a significant worldwide public health crisis, impacting about 537 million individuals globally [1]. The majority of this global population, approximately 80%, resides in low- and middle-income countries (LMICs), where the prevalence of diabetes is rising most rapidly [2]. Diabetes is a rising cause of death and, in 2020, was noted to rank within the top ten causes of mortality in LMICs [3]. Diabetes is also responsible for dire and disabling complications such as cardiovascular disease, stroke, neuropathy, nephropathy, and diabetic retinopathy (DR), a major cause of blindness worldwide [3-5].

As the global prevalence of diabetes continues to surge, especially within LMICs [2], it underscores the critical need for proactive measures such as early detection, timely treatment, and vigilant follow-up to reduce the risk of vision impairment and blindness. The disproportionate increase in populations with diabetes and diabetic-related health complications in LMICs is exacerbated by the disparity in the distribution of ophthalmologists globally. In 2015, the mean ophthalmologist density in LMICs was reported to be approximately 3.7 per one million persons, whereas, in high-income countries (HICs), the mean density was considerably higher at approximately 76.2 per one million persons, representing an 18-fold difference [6]. Limited access to skilled eye specialists, practitioners, and facilities leads to delayed diagnosis and intervention of DR, causing the progression of sight-threatening diabetic retinopathy (STDR) and eventually irreversible blindness [7]. Furthermore, the chronicity of diabetes and the long-term complications associated with undiagnosed and untreated diabetes not only impact a patient’s standard of living but also impose a substantial financial strain on patients, their families, and the healthcare system [8]. In 2021, diabetes was responsible for an estimated 966 billion United States dollars (USD) in global health expenditure, up from the 232 billion USD spent on diabetes care in 2007 [1]. This 316% increase in expenditure over the last 15 years has most directly impacted and burdened the healthcare systems of LMICs [1,9,10].

With the exponential rise in diabetes, individuals in LMICs are left facing a series of challenging issues, highlighting the crucial need for novel solutions for DR screening. While artificial intelligence (AI) is being used in various medical fields such as pathology, radiology, and cardiology to improve diagnostic accuracy [11], the development of AI-based methods for the diagnosis and detection of various ophthalmic diseases has significantly increased due to the widespread accessibility and availability of ophthalmic imaging [12]. Available automated algorithms may offer the promising potential to increase the accuracy of detecting early-stage DR, enable timely interventions to improve patient outcomes and reduce health disparities by increasing accessibility in remote or underserved areas [13]. Although AI-based algorithms have received regulatory authorization for DR detection in the United States, Europe, and Singapore [14], a unified AI system has yet to be implemented into clinical practice. Considerable research gaps exist concerning the ethical, legal, and economic considerations related to the clinical integration of AI algorithms [15]. The specific aims of this systematic review are to: summarize the impact of the cost of diabetes globally; examine the cost implications of AI implementation for DR screening in LMICs; and guide ophthalmologists, eye care professionals, researchers, and policymakers in the decision-making process for potentially implementing AI into healthcare systems.

## Review

Methods

Data Sources

**Figure 1:**
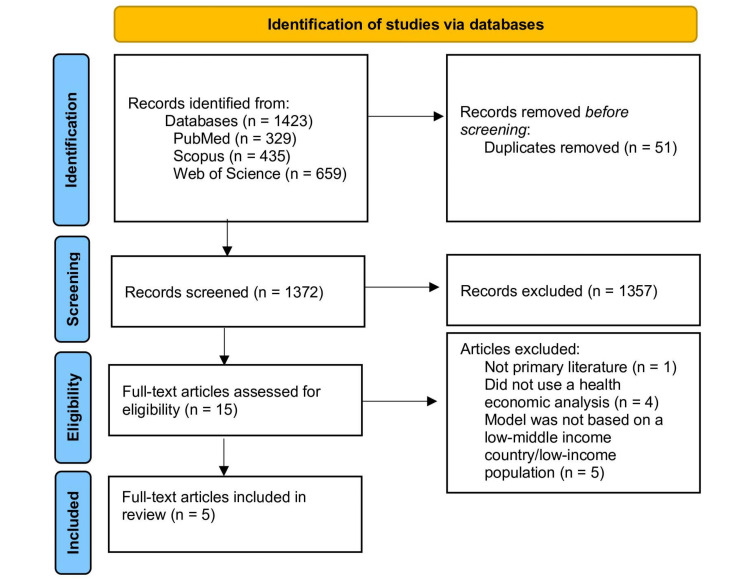
PRISMA flow diagram outlining the screening and selection process of articles obtained from PubMed/MEDLINE, Scopus, and Web of Science databases PRISMA: Preferred Reporting Items for Systematic Reviews and Meta-Analyses

This review adhered to the Preferred Reporting Items for Systematic and Meta-Analyses (PRISMA) guidelines [16]. Given the nature of a systematic review, we did not require Institutional Review Board approval. A systematic review of the literature was performed in PubMed/MEDLINE, Scopus, and Web of Science on July 15, 2023, to identify peer-reviewed articles (Figure 1). In consultation with a research librarian, we selected search terms that were a combination of Medical Subject Headings (MeSH) and keywords (Table 1). We used Reference Manager to manage the identified records.

**Table 1:** Search string used in the database search for the systematic literature review

Database	Search String				
PubMed	“ophthalmology” OR "diabetic retinopathy" OR "nonproliferative diabetic retinopathy" OR "proliferative diabetic retinopathy" OR "Diabetic Retinopathy"[Mesh]	AND	"artificial intelligence" OR "deep learning" OR "automated screening" OR "fundus photograph" OR "fundus image" OR "machine learning" OR "fundus images" OR "fundus photographs" OR "Artificial Intelligence"[Mesh] OR "Deep Learning"[Mesh] OR "Machine Learning"[Mesh]	AND	“cost-utility analysis" OR "cost-effectiveness analysis" OR "cost-benefit analysis" OR "cost-minimization analysis" OR "economic evaluation" OR “cost” OR "healthcare economics" OR "Cost-Benefit Analysis"[Mesh] OR "Cost-Effectiveness Analysis"[Mesh]
Scopus	“ophthalmology” OR "diabetic retinopathy" OR "nonproliferative diabetic retinopathy" OR "proliferative diabetic retinopathy"	AND	"artificial intelligence" OR "deep learning" OR "automated screening" OR "fundus photograph" OR "fundus image" OR "machine learning" OR "fundus images" OR "fundus photographs"	AND	"cost-utility analysis" OR "cost-effectiveness analysis" OR "cost-benefit analysis" OR "cost-minimization analysis" OR "economic evaluation" OR “cost” OR "healthcare economics"
Web of Science	“ophthalmology” OR "diabetic retinopathy" OR "nonproliferative diabetic retinopathy" OR "proliferative diabetic retinopathy"	AND	"artificial intelligence" OR "deep learning" OR "automated screening" OR "fundus photograph" OR "fundus image" OR "machine learning" OR "fundus images" OR "fundus photographs"	AND	"cost-utility analysis" OR "cost-effectiveness analysis" OR "cost-benefit analysis" OR "cost-minimization analysis" OR "economic evaluation" OR “cost” OR "healthcare economics"

Study Selection

All titles and abstracts were separately screened by two reviewers (AR and FR) for relevance to the research question. Results from the screening process were compared, and a total list of eligible articles as identified by either one or both reviewers was developed. Then, both reviewers (AR and FR) performed parallel independent assessments of the complete text articles to ascertain that the articles met the specified criteria for inclusion and exclusion. Any disagreements were solved by adjudication with another author (AVD). Our inclusion criteria were articles that were: (i) primary research articles; (ii) had full-text viewable online and were in English; (iii) discussed the economic evidence of AI-based DR screening using a health economic analysis (HEA); and (iv) used an LMIC or low-income population/setting. LMICs were categorized based on the 2022 World Bank classification [17]. We excluded articles that did not use economic model analyses and only alluded to or briefly discussed the potential economic costs of AI-based DR screening. Articles were used and discussed in the context of an LMIC.

Data Extraction

Information was extracted by two reviewers (AR and FR) using an agreed-upon data extraction template. Data were extracted from each study regarding the author, screening and comparison models, HEA, outcome measures, and strengths and weaknesses as identified within the paper by the authors of the study.

Bias Assessment

Utilizing the Joanna Briggs Institute (JBI) critical appraisal checklist for economic evaluations, two reviewers (AR and FR) independently evaluated the methodological rigor and potential bias in each study. The JBI critical appraisal checklist does not include a scoring system, and therefore, both reviewers discussed and arrived at a consensus on the cumulative risk of bias for each article.

Results

Our search identified a total of 1423 results: 329 results on PubMed/MEDLINE, 435 results from Scopus, and 659 results from the Web of Science (Figure 1). Then, 1372 titles and abstracts were screened after the removal of 51 duplicates. After screening titles and abstracts, fifteen articles underwent full-text review. A total of five articles were identified for final inclusion. Based on the criteria in the JBI critical appraisal checklist for economic evaluations, three articles had a low risk of bias, and two articles had a moderate risk of bias.

Health Economic Analyses

The most commonly used HEAs include cost-effectiveness analysis (CEA), cost-utility analysis (CUA), cost-minimization analysis (CMA), and cost-benefit analysis (CBA) [18]. The studies incorporated in our systematic review employed CEAs (three studies) and CUAs (three studies) (Table 1). A CEA is a HEA that compares the expenses and health outcomes of different interventions, programs, and treatments. The primary goal of a CEA is to evaluate the economic costs of various interventions and determine if the value of an intervention justifies its cost by considering both the costs incurred and outcomes achieved [18,19]. A CUA assesses the cost-effectiveness of various interventions with distinct outcomes by evaluating their utility using a standardized measure [18].

The results from these HEAs will be summarized below in terms of quality-adjusted life years (QALYs) and cost ratios. In a CUA, patient outcomes are measured by QALYs, which evaluate the impact of interventions on the health and well-being of an individual [20]. When interpreting QALYs in a HEA, a higher number of QALYs associated with an intervention indicates an improvement in the patient’s quality of life or length of life [21]. Incremental cost-effectiveness ratio (ICER) is a crucial measure that assesses the efficiency of an intervention in terms of costs and benefits and compares the additional cost of one intervention over another to the additional benefits, which are often measured in QALYs gained. When interpreting ICER values, a lower ICER value suggests that the intervention is providing more health benefits for every additional unit of cost compared to the alternative. ICER values are compared against a predetermined threshold to determine cost-effectiveness. When the ICER value falls beneath the predefined threshold, the intervention is deemed to be cost-effective [22].

Cost-Effectiveness Analyses

Three out of the five articles incorporated in our systematic review used a CEA as their HEA model. One study (Huang et al.) evaluated the cost-effectiveness of utilizing AI-based screening to detect DR in a group of 1,000 diabetic patients residing in rural China. The study compared AI-based screening to no screening and screening conducted by ophthalmologists, in which fundus images were evaluated and graded. This study included two different viewpoints: a health system perspective and a societal perspective. The evaluation of effectiveness was conducted by measuring the increase in QALYs. Results revealed that from a health system perspective, compared to no screening, AI screening had a higher cost of $180.19 and an increase of 0.16 in QALYs. Relative to AI screening, ophthalmologist screening incurred greater expenses with a cost of $215.05 and was found to be less effective with a decrease of 0.04 in QALYs. From a societal perspective, AI screening costs less ($1,683.23) compared to ophthalmologist screening ($1,775.48). Relative to AI screening, ophthalmologist screening incurred greater expenses and was found to be less effective, with a decrease of 0.04 in QALYs. Compared to no screening, the ICER for AI screening was $10,347.12, which was lower than the threshold needed to be considered cost-effective. Ultimately, the study concluded that, from a health system viewpoint and a societal viewpoint, AI-based screening is more economically efficient than both traditional ophthalmologist screening and no screening for DR [23].

A second study by Lin et al. performed both a CEA and a CUA to understand if the use of an AI model compared to manual grading maintains a comparable level of cost-effectiveness. In the study, using urban China as a model, 15,663 individuals were screened with AI, and 17,032 individuals were screened with manual grading. Results showed that the total cost for a community resident with diabetes in the AI-supported screening method was 2.5% less compared to the manual-grading screening method, but it resulted in a reduction of 0.3% in the number of years without blindness and a decrease of 0.1% in QALYs. The ICER for the AI-supported approach was US $2,553.39 per year without blindness, suggesting that it was not economically viable. Further sensitivity analysis indicated that if compliance with referrals rises by 7.5% following AI implementation, costs of on-site screenings using the manual-grading approach increase by 50%, or costs of on-site screenings using the AI-based approach decrease by 50%, then the AI-assisted DR telemedicine screening approach could be considered the more advantageous choice. The acceptability curve suggested that the manual-grading screening strategy was the preferred approach in 60.6% of the simulations when the threshold was established at the gross domestic product (GDP) per capita (US $22,600). Furthermore, the manual-grading screening strategy was favored in 84.5% of the simulations when the threshold was set at US $67,800 [24].

A third study by Gomez Rossi et al. performed a CEA of AI for the detection of DR. In the study, the model included a group of individuals aged 40 years or older with type 2 diabetes mellitus (T2DM) who were at risk for developing DR. For AI-assisted screening, the mean cost was Brazilian real (R) $1,321, while the standard ophthalmologist screening cost was R $1260.28. Both AI-assisted screening and standard ophthalmologist screening resulted in a nearly identical average utility of 8.4 QALYs; however, the AI-assisted screening strategy incurred an additional cost of R $61. The ICER was R −$91,760, suggesting that AI-assisted screening relative to standard screening yielded a higher cost with no significant gain in QALYs. The cost-effectiveness acceptability curve suggested that compared to AI-assisted screening, the standard of care was more cost-effective; however, as the willingness to spend more money to achieve better outcomes (e.g., QALYs) increased, there was an increased level of uncertainty about which strategy would be more cost-effective. According to the World Health Organization's (WHO) recommendations, a maximum threshold of R $43,689 for each QALY gained was deemed cost-effective. Given that the incremental cost per QALY with AI-assisted screening (R $39,705) was below the threshold, AI screening was considered cost-effective by the WHO's guidelines [25].

Cost-Utility Analyses

One study by Fuller et al. performed a CUA of two different screening approaches: automated retinal image analysis system (ARIAS)-based DR screening versus an annual dilated screening eye examination. The economic modeling analysis was conducted in the setting of a primary care clinic and used low-income patients with diabetes who were at least 18 years old as its cohort. Results showed that over a five-year period, ARIAS had a cost of $1,596.99 and yielded 4.942 QALYs, resulting in a cost-utility ratio of 323.1 ($1,596.99/4.942). The current practice had a cost of $2,082.91 and yielded 4.944 QALYs, resulting in a cost-utility ratio of 421.3 ($2,082.91/4.944). When comparing ARIAS to the current practice, ARIAS was associated with substantial cost savings (a 23.3% decrease in USD) and nearly comparable utility (a 0.04% decrease). While the measured primary outcome of an ICUR of $258,721.81 suggested that ARIAS implementation may still require a substantial investment to gain one additional QALY compared to the current practice, the cost savings it provided were significant (P < .001). Fuller et al. showed that the cost reduction was likely attributed to several factors, such as the fact that 59.4% of patients with vision-threatening diabetic retinopathy (vtDR) were more likely to adhere to follow-up ophthalmic recommendations determined by ARIAS technology in comparison to the current practice (33.8% adherent). Improved compliance with follow-up recommendations allows for timely interventions, preventing not only further vision complications but also the increase in the cost of care associated with increased disease progression and severity. From years two to five, treatment costs for patients with proliferative diabetic retinopathy (PDR) with diabetic macular edema were shown to be increasingly higher ($6,614.06) than those for patients with only PDR ($2,661.16), representing the cost-benefit of early intervention. Ultimately, the study concluded that AI screening was more cost-effective than the conventional dilated screening eye examination [26].

Research conducted by Lin et al. examined the economic implications of adopting AI for telemedicine-based DR screening in urban China. The study utilized both a CEA and a CUA to analyze the costs and effectiveness of AI-based DR screening methods in comparison to the current standard of manual grading of retinal images. The AI-based DR screening model offered a 2.5% reduction in cost ($3,182.50) when compared to the manual-grading model ($3,265.40) and nearly comparable QALYs of 6.748 and 6.753, respectively. The main outcome measured in this study was the ICUR. The ICUR value of $15,216.96 indicated that AI-based DR screening was not more cost-effective than manual-grading screening methods in a setting such as Shanghai. The paper defined that interventions reducing the participants’ utilities needed to have cost savings above the threshold of US $22,600 (GDP per capita) to be deemed cost-effective [24].

Lastly, a CUA done by Srisubat et al. investigated the economics of a DR screening initiative using deep learning (DL) technology compared to skilled human graders (HG). Srisubat et al. used Thailand for their model and included individuals greater than 40 years old with T2DM who received an annual screening via either DL or HG. From a societal perspective, DR screening using DL had a total cost of $163,478.16 Thai baht (THB), whereas screening using trained HG had a cost of $163,565.04 THB. Between the two different screening methods, there was an incremental cost difference of 86.88 THB, indicating the DL-based DR screening method has a cost reduction of approximately 0.05%. Both methods offered comparable QALYs of 12.8617 and 12.8574 for DR screening using DL and human graders, respectively. The study also examined the influence that adherence to treatment recommendations may have on determining the cost-effectiveness of the two screening methods. The results indicated that even if the rate of compliance with treatment referrals was lower for DL (44% compliance) than for HG (60% compliance), the DL screening method would still be cost-effective [27].

The results from the five articles (Table 2) and the JBI critical appraisal checklist for economic evaluations (Table 3) are summarized below.

**Table 2:** Summary of currently available literature on health economic analyses of artificial intelligence implementation for diabetic retinopathy in low- and middle-income countries AI: artificial intelligence, ARIAS: automated retinal image analysis system, DR: diabetic retinopathy, CEA: cost-effectiveness analysis, CUA: cost-utility analysis, CBA: cost-benefit analysis, CMA: cost-minimization analysis, ICER: incremental cost-effectiveness ratio, ICUR: incremental cost-utility ratio, QALY: quality-adjusted life year, DL: deep learning, HG: human grader, GDP: gross domestic product, LY: life years, THB: Thai baht, USD: United States dollar, R$: Brazilian real, WTP: willingness-to-pay

Reference	HEA/model	Models and Comparators	Primary Outcome Measure	Results	Strengths	Limitations
Huang et al. [23]	CEA/Markov model-based hybrid decision tree	AI screening was compared with ophthalmologist screening, in which fundus images were evaluated by ophthalmologists	ICER	Health system perspective- Relative to no screening, AI screening was more expensive with a cost of $180.19 but more effective with an incremental QALYs of 0.16. The ICER of the AI screening group compared with the no screening group was $1,107.63/QALY gained, less than the threshold of $30,765.09. Societal perspective- AI screening costs less than ophthalmologist screening ($1,683.23 versus $1,775.48). Relative to no screening, the ICER of AI screening was $10,347.12, below the cost-effective threshold $30,765.09.	Applied a more comprehensive system of prognosis after people were diagnosed with diabetes. In the study, health states included DR states, blindness, death, and the stable state after laser treatment, which reflect the natural progression of DR.	The utility values were partly derived from the results in other countries, which might not be exactly consistent with those in China. The ICER was compared with the per capita GDP of the whole country instead of rural China.
Lin et al. [24]	CEA and CUA/decision-analytic Markov model	AI screening was compared to manual-grading	ICER and ICUR	In the manual grading–based telemedicine screening, the total cost was US $3,265.40 with 9.83 years without blindness and 6.753 QALYs. In the AI-based telemedicine screening, the total cost was US $3,182.50, with 9.80 years without blindness and 6.748 QALYs. ICUR was US $15,216.96 per QALY and ICER was US $2,553.39.	Conducted a sensitivity analysis within wide ranges and identified the most influential variables affecting the decision to use AI and manual grading in telemedicine screening.	Mainly based on empirical data from Shanghai; therefore, it cannot be representative of all of China because of the huge regional and medical care differences between urban and rural areas.
Gomez Rossi et al. [25]	CEA/Markov model	AI screening was compared to standard screening of DR undertaken by ophthalmologists	Association of AI with QALYs	The mean cost was R $1,321 for AI and R $1,260.28 for diagnosis without AI. Both strategies yielded a very similar mean utility of 8.4 QALYs. The ICER was US R $-91,760. The acceptability curve showed that standard of care was more likely to be more cost-effective although higher WTP increased the uncertainty about the optimal strategy.	The main strength was its design, which modeled different AI technologies for detecting three different diseases such as melanoma, dental caries, and DR, and compared them against established medical practices.	Limited information available on the research, operation and overhead costs, and payment mechanisms involved in incorporating AI did not allow for generating detailed comparisons.
Fuller et al. [26]	CUA/Markov model	ARIAS-based DR screening was compared to standard, in-office dilated eye examinations	ICUR	A 23.3% reduction in cost (USD) in the ARIAS group compared with the standard practice (P < .001). Comparing the current practice to ARIAS screening, an ICUR of $258,721.81 was calculated, which was well beyond the assessed willingness-to-pay threshold of $100,000.	Results were analyzed over a five-year period.	Model only included direct costs of screening and treatment from the payor’s perspective and did not account for indirect costs to patients.
Srisubat et al. [27]	CUA/decision tree-Markov hybrid model	DL screening was compared to human graders	Total cost incurred by the health care system and the total QALYs gained per patient	From societal and provider perspectives, there was equal effectiveness in LY for HG and DL at 18.53, whereas QALYs were 12.857 and 12.862, respectively. From a societal perspective, DL cost was 163,478.16 THB vs. HG cost was 163,565.04 THB, representing an 87 THB cost difference in favor of the DL strategy. From a provider perspective, DL was found to have a higher incremental cost at 2,195 THB and the ICER was 512,955 THB.	Evaluated cost-savings over a lifetime horizon of patients.	Model did not consider the possibility that DL may flag more ungradable patients than HG in real-world scenarios, and therefore, false negatives were not accounted for.

**Table 3:** Joanna Briggs Institute critical appraisal checklist for economic evaluations JBI: Joanna Briggs Institute

JBI—Critical Appraisal Checklist for Economic Evaluations	Huang et al. [23]	Lin et al. [24]	Gomez Rossi et al. [25]	Fuller et al. [26]	Srisubat et al. [27]
Is there a well-defined question?	Yes	Yes	Yes	Yes	Yes
Is there a comprehensive description of alternatives?	Yes	Yes	Yes	Yes	Yes
Are all important and relevant costs and outcomes for each alternative identified?	Yes	Yes	No	No	No
Has clinical effectiveness been established?	Yes	Yes	Yes	Yes	Yes
Are costs and outcomes measured accurately?	Unclear	Unclear	Unclear	Unclear	Yes
Are costs and outcomes valued credibly?	Unclear	Yes	Yes	Yes	Yes
Are costs and outcomes adjusted for differential timing?	No	No	Unclear	Yes	No
Is there an incremental analysis of costs and consequences?	Yes	Yes	Yes	Yes	Yes
Were sensitivity analyses conducted to investigate uncertainty in estimates of cost or consequences?	Yes	Yes	Yes	Yes	Yes
Do study results include all issues of concern to users?	Unclear	Unclear	Unclear	Unclear	Unclear
Are the results generalizable to the setting of interest in review?	Unclear	Yes	Unclear	No	Yes
Overall Rating	Moderate risk	Low risk	Moderate risk	Low risk	Low risk

Discussion

Our comprehensive systematic review is the first to summarize the worldwide literature concerning the HEAs of AI-based methods for DR screening in LMICs, with a focus on how such studies may be adaptable to India. As AI continues to evolve, it is clear that despite advancements being made, there is still more to understand, as our systematic review identified only five studies. In our systematic review, three out of the five studies showed that AI is more economically efficient than traditional screening approaches for DR. The studies evaluated economic evidence from China, Thailand, and Brazil, but there was no primary literature from India. Our systematic review suggests that more primary HEAs are necessary to determine the economic evidence for the adoption of AI systems for DR screening in India.

An Indian Perspective

T2DM, which comprises 90% of diabetes cases, bears a substantial burden in India, with India ranking second after China in the global diabetes epidemic [28]. The 2019 International Diabetes Federation (IDF) Atlas reported approximately 77 million people in India had T2DM, deeming India the "diabetes capital of the world" [1,29]. In the upcoming years, projections of individuals with T2DM in India are expected to reach 101 million by 2030 and 134.2 million by 2045 [28]. Compared to the Western population, India has traditionally seen the age of onset of T2DM one to two decades earlier, placing patients at an increasingly higher risk of developing diabetic-related complications [30]. As the prevalence of early-onset T2DM rises, nearly 57% of people with diabetes in India remain undiagnosed and unaware of their condition, representing the critical importance of improved screening and awareness [28].

DR is a progressive disease that spans a spectrum of severity, ranging from non-sight-threatening diabetic retinopathy (NSTDR) to sight-threatening diabetic retinopathy (STDR) [31]. In India, a tertiary care facility found that increased costs are associated with more advanced conditions such as STDR, with the cost of care for people afflicted by STDR double that of individuals without such severe retinopathy (31,820 Indian rupees (INR); 430 USD versus 14,356 Indian rupees (INR); 194 USD; P < 0.001). Increased costs associated with STDR were due to several different factors, including an increased number of intravitreal injections, retinal laser procedures, visits to hospitals, and presumed medication prescriptions [32]. The direct correlation between increased costs and progressive disease severity underscores the critical need for enhanced screening methods and prevention strategies. Results from our systematic review underscore the existing scarcity of economic evidence surrounding the implementation of AI for DR screening in LMICs, highlighting a need for further research to investigate if AI is a cost-effective screening solution that is capable of alleviating financial strain linked to prolonged complications arising from diabetes.

Furthermore, a notable factor when considering the implementation of AI-based DR screening in India is the prevalence of a predominantly rural population. In 2022, approximately 1.4 billion people lived in India, with 64% of the total population living in rural areas [33]. While a majority of the Indian population lives in rural areas and villages, these areas are the ones experiencing the greatest spatial inequalities in terms of the allocation of healthcare investments, medical facilities, medical professionals, and transportation connectivity [34,35]. For instance, in 2016, there were only 11 ophthalmologists serving one million people in India [36], and nearly 70% of ophthalmologists resided in urban areas, making access to eye care extremely challenging for residents in rural areas [37]. Ambade et al. found that in India, the burden of nonmedical costs (transportation, lodging, etc.) was higher among those who are rural residents, of low-income status, and less educated. Poor health infrastructure and a lack of access to testing and basic care were identified as leading reasons for higher indirect costs for these groups [38]. Introducing AI-based DR screening methods in India has the potential to reduce barriers to access [13], alleviating associated health expenditures and the strain of nonmedical costs for minority groups.

Limitations

It is important to acknowledge the various potential limitations associated with this systematic review. Firstly, while the healthcare systems of China, Thailand, and Brazil may share some similarities with the Indian healthcare system, there are notable differences that limit the practicality of making broad generalizations, as we need to consider that other LMICs will also have differing challenges. Direct labor costs in HICs such as Singapore and the United Kingdom have been carefully evaluated [39,40]. However, an accurate assessment of expenses related to human resources in India has not been conducted, so conclusions drawn from HICs or even other LMICs might not be equally applicable. Additionally, indirect costs to patients (i.e., income loss due to blindness, transportation costs, etc.) vary significantly among different regions, locations, and internationally. Furthermore, the articles analyzed in our systematic review primarily address the favorable and promising cost-effectiveness of employing AI for DR screening. However, the limited primary literature evaluating the negative consequences, such as misdiagnoses, patient privacy concerns, possible displacement of jobs, etc., as well as the economic impact of implementing AI-based methods for DR screening, raises concern for potential publication bias. Lastly, our systematic review only included studies that were published in English, which is of particular significance as English is not the primary language of many LMICS. As a result, critical and relevant studies in foreign languages may have been excluded.

Future research

The potential of AI in clinical practice holds promising opportunities, yet further research is essential to fully explore its capabilities, refine its applications, and ensure its seamless integration into clinical care workflows. First, AI can be employed to extensively delve into the realm of data exploration, revealing novel patterns and relationships through advanced data mining techniques. Additionally, the utilization of AI in evaluating and examining prior decisions can be achieved through methodologies such as randomized controlled experiments and multivariate testing, ensuring a comprehensive understanding of their outcomes and results [41]. Furthermore, AI’s potential can be harnessed to elucidate the underlying causal factors behind specific outcomes and results, employing statistical and descriptive analysis to offer insightful explanations. Lastly, AI’s capacity extends to forecasting future outcomes, enlisting predictive modeling and analytics to explore unfamiliar territories and enable informed decision-making [42]. Across all of these aspects, AI emerges as a pivotal and crucial tool with the unique potential to reshape research paradigms and offer profound insights across diverse domains.

Various efforts to develop AI algorithms to eliminate preventable blindness and massively scale the delivery of eye screenings have been underway in India. In a study conducted at Aravind Eye Hospital and Sankara Nethralaya Eye Center, Gulshan et al. showed that an automated DR grading system performed equally or better than manual grading of retinal fundus photographs [43]. While there is active research into the development and performance accuracy of AI for DR detection, it is important to acknowledge the clear research gap that exists from developing to implementing AI systems in clinical practice. In particular, LMICs will face unique challenges in the clinical implementation of AI, highlighting the need for more research on AI in low-resource settings. As many LMICs face a shortage of ophthalmologists and a lack of medical facilities and infrastructure to support the growing population that will require prompt treatment and care after AI referral, the utility of leveraging AI technologies to improve health outcomes may not be attainable until these disparities in access to care are solved.

There are multiple areas for future research that warrant further investigation, such as the ethics, privacy, legality, and economic impact of AI use in healthcare [44-48]. Accurate economic evaluations demand context-specific data that accounts for the unique socio-economic and healthcare landscape of regions such as India. Specifically, future research that focuses on a comprehensive understanding of the direct, indirect, and human resource costs related to DR in India is needed to help assess the cost-effectiveness of novel AI technologies against the standard of care.

## Conclusions

Currently, there is limited literature regarding the HEAs of AI-based DR screening methods in LMICs. Specifically, our systematic review showed that no such study in the context of India exists. The economic evidence for the integration of AI algorithms for DR screening exhibits notable heterogeneity due to variations in treatment expenses, direct and indirect costs, and healthcare systems across LMICs. Although our systematic review suggests the economic feasibility of AI for DR screening in China and Thailand, these positive results cannot be directly extrapolated to the Indian setting. In conclusion, our systematic review underscores the need for further research and economic assessments to enhance our understanding of the economic implications of implementing AI in clinical practice and help design effective public health strategies tailored to address the challenges posed by diabetes.
